# On the Capacity of 5G NR Grant-Free Scheduling with Shared Radio Resources to Support Ultra-Reliable and Low-Latency Communications

**DOI:** 10.3390/s19163575

**Published:** 2019-08-16

**Authors:** M. Carmen Lucas-Estañ, Javier Gozalvez, Miguel Sepulcre

**Affiliations:** Department of Communications Engineering, Universidad Miguel Hernández de Elche (UMH), Avda. de la Universidad s/n, 03202 Elche, Spain

**Keywords:** grant-free, scheduling, URLLC, ultra-reliable and low-latency communications, 5G, deterministic, time-critical, reliability, latency, aperiodic traffic, Industry 4.0

## Abstract

5G and beyond networks are being designed to support the future digital society, where numerous sensors, machinery, vehicles and humans will be connected in the so-called Internet of Things (IoT). The support of time-critical verticals such as Industry 4.0 will be especially challenging, due to the demanding communication requirements of manufacturing applications such as motion control, control-to-control applications and factory automation, which will require the exchange of critical sensing and control information among the factory nodes. To this aim, important changes have been introduced in 5G for Ultra-Reliable and Low-Latency Communications (URLLC). One of these changes is the introduction of grant-free scheduling for uplink transmissions. The objective is to reduce latency by eliminating the need for User Equipments (UEs—sensors, devices or machinery) to request resources and wait until the network grants them. Grant-free scheduling can reserve radio resources for dedicated UEs or for groups of UEs. The latter option is particularly relevant to support applications with aperiodic or sporadic traffic and deterministic low latency requirements. In this case, when a UE has information to transmit, it must contend for the usage of radio resources. This can lead to potential packet collisions between UEs. 5G introduces the possibility of transmitting *K* replicas of the same packet to combat such collisions. Previous studies have shown that grant-free scheduling with *K* replicas and shared resources increases the packet delivery. However, relying upon the transmission of *K* replicas to achieve a target reliability level can result in additional delays, and it is yet unknown whether grant-free scheduling with *K* replicas and shared resources can guarantee very high reliability levels with very low latency. This is the objective of this study, that identifies the reliability and latency levels that can be achieved by 5G grant-free scheduling with *K* replicas and shared resources in the presence of aperiodic traffic, and as a function of the number of UEs, reserved radio resources and replicas *K*. The study demonstrates that current Fifth Generation New Radio (5G NR) grant-free scheduling has limitations to sustain stringent reliability and latency levels for aperiodic traffic.

## 1. Introduction

5G networks are being designed with the objective to support a broad range of verticals such as manufacturing, transport, health, energy and entertainment. To this aim, important changes have been introduced to increase data rates (enhanced mobile broadband, or eMBB), efficiently support large amounts of devices (massive machine type communications, or mMTC) and guarantee unprecedented reliability and latency levels (Ultra-Reliable and Low-Latency Communications or URLLC) [1]. Supporting URLLC is particularly relevant for many Industry 4.0 manufacturing applications, such as motion control (requires a maximum latency of 1 ms and a reliability of 1–10^−6^ [2]), control-to-control applications (maximum latency of 4 ms and a reliability of 1–10^−8^ [1]) and factory automation (maximum latency between 0.25 ms and 2.5 ms and reliability requirements up to 1–10^−9^ [3]). These applications require the exchange of information between sensors, actuators and controllers through an industrial sensor and control network. 5G has the potential to provide the connectivity required by the Industry 4.0 to digitalize factories and to support data-intensive services while ubiquitously guaranteeing low latency and reliable connections. This has actually been acknowledged through the establishment of the 5G Alliance for Connected Industries and Automation (5G-ACIA) [4].

5G has introduced significant changes to support URLLC [5]. Some of these changes focus at the Radio Access Network level, since the medium access mechanisms account for an important part of the total end-to-end transmission delay [6]. This is for example the case of the grant-based scheduling process for uplink (UL) transmissions in legacy LTE (Long Term Evolution) 4G networks. Grant-based scheduling requires a User Equipment (UE) and a Base Station (BS) to exchange scheduling requests (SRs) and grant messages before transmitting any data. This process alone already results in an average delay of up to 11.5 ms when considering a Transmission Time Interval (TTI) equal to 1 ms and an SR periodicity of 10 ms [3]. Reducing the slot duration can reduce this delay. However, additional scheduling changes have been necessary to sustain the URLLC requirements that characterize some vertical applications, such as those in Industry 4.0. In particular, Release 15 and 16 of the 3rd Generation Partnership Project (3GPP) standards have introduced the concept of grant-free scheduling (also referred to as Configured Grant for 5G New Radio [7]) to support URLLC.

With grant-free scheduling, the BS reserves resources for UL transmissions and informs the UEs of the reserved resources. When a UE wants to initiate a UL transmission, it directly utilizes the reserved resources, without sending an SR and waiting for the subsequent grant message from the BS. Recent studies have shown that grant-free scheduling in 5G NR considerably reduces the end-to-end latency [8]. The 3GPP standards introduce the possibility for grant-free scheduling to reserve resources to dedicated UEs, or to a group of UEs. In the first case, each resource is reserved for a specific UE, and only this UE can utilize the resource at any time. This approach is adequate for periodic traffic since the resource allocations can be planned, and resources can then be utilized efficiently. Such planning is not possible in the case of aperiodic, sporadic or uncertain traffic. Sharing dedicated resources by a group of UEs is hence an interesting option to optimize the usage of the radio resources in the presence of aperiodic traffic. In this case, UEs have to contend for their usage, and collisions are possible. 5G NR introduces the possibility to transmit *K* replicas of the same packet in consecutive slots to combat potential collisions. However, relying on the transmission of *K* replicas to achieve a target reliability level can result in additional delays. It is yet unknown whether 5G NR grant-free scheduling with *K*-repetitions and shared resources can satisfy critical applications and guarantee very high reliability levels with very low latency. In this context, this study presents an in-depth analysis of the reliability and latency levels that can be achieved with existing 5G NR grant-free scheduling solutions as a function of the number of UEs, the number of reserved radio resources, and the number of replicas *K*. To this aim, the study analytically quantifies the probability of successfully delivering a packet when using grant-free scheduling with *K*-repetitions and shared resources. In addition, the study analyzes the impact of self-collisions. Self-collisions occur when a UE has to transmit a new packet, and the transmission of the *K* replicas of the previous packet has not finished. If this happens, the new packet must be stored, and its transmission is delayed until all replicas of the previous packet have been transmitted. This study demonstrates for the first time that self-collisions have a non-negligible impact upon the capacity of 5G NR grant-free scheduling to support stringent URLLC reliability and latency levels.

## 2. Related Work

The 5G NR standard introduces the use of grant-free scheduling (also referred to as Configured Grant [7]). With grant-free scheduling, the network pre-configures the radio resources and assigns them to UEs without waiting for UEs to request resources. UEs can utilize the pre-assigned resources as soon as they have data to transmit. This is in contrast to grant-based scheduling, where UEs must request access to radio resources through the transmission of Scheduling Requests (SR). The BS assigns the radio resources to the UEs and notifies them using grant messages. UEs must wait to receive these grant messages before transmitting any data. Grant-free scheduling eliminates all delays introduced by the handshaking present in grant-based scheduling. Grant-free scheduling also improves the energy consumption of the UEs, reduces their complexity, and decreases the signaling overhead compared with grant-based scheduling ([8,9]). Grant-free scheduling can assign dedicated or shared resources to the UEs. The BS decides whether resources are dedicated to specific UEs, or are shared by a group of UEs [10]. Reserving resources to dedicated UEs is an interesting approach when we can plan ahead what is the demand for resources. This is for example the case of periodic traffic. However, reserving resources to dedicated users can be highly inefficient if the traffic demand is uncertain or aperiodic, and it is not possible to anticipate when these resources will be needed. In this case, it is possible to share radio resources by a group of UEs. This option ensures a more efficient utilization of resources, and the possibility to satisfy URLLC communication requirements. However, users must contend for the resources, and collisions can happen if two or more UEs simultaneously contend for the same resources. 5G NR introduces the possibility of transmitting *K* replicas of the same packet in consecutive slots to combat collisions and thus increase the probability of a correct reception [11,12].

The study in [13] analyzes the performance of the *K* replicas scheme. The authors propose transmitting the first copy of a packet using dedicated resources, and the following replicas using shared resources. The proposal also exploits shared diversity and advanced receiver processing techniques to reduce the impact of packet collisions. The proposal achieves adequate reliability levels and reduces the number of reserved (shared) radio resources, compared to a configuration that reserves resources to dedicated UEs. The study in [14] also transmits the first copy of a packet using dedicated resources. However, it does not consider the transmission of *K* replicas of a packet. Instead, the authors propose to retransmit the original packet in a shared resource only if the first transmission is not successful. This requires a handshaking between the UEs and the BS to exchange acknowledgement messages. This handshaking increases the latency, and can compromise the capability to adequately support URLLC applications with stringent latency requirements. In [15], the authors study the optimum number of replicas (*K*) necessary to achieve a target reliability level within a deterministic latency deadline. The study focuses upon aperiodic traffic and the case in which a group of UEs share resources. The authors show that randomly choosing the resource for each replica increases the probability of correctly delivering a packet. However, the study focuses on reliability levels up to 1–10^−5^ while some critical Industry 4.0 applications require higher reliability levels.

Previous studies have shown that transmitting *K*-repetitions of a packet increases the reception rate. However, this can be done at the expense of an inefficient use of the radio resources due to packet collisions or the unnecessary reservation of resources when the first replicas are correctly delivered. Latency requirements may also impose restrictions on the number of replicas that can be transmitted, and consequently on the reliability levels that may be achieved. In this context, several recent contributions have analyzed slight modifications to the *K*-repetitions scheme. For example, [16] proposed adaptively configuring the number of replicas transmitted based on the channel conditions. The objective is to utilize the radio resources efficiently by avoiding unnecessary retransmissions when the channel quality is good. A similar objective is sought in [17] where authors propose conditions to stop the transmission of replicas. Other interesting proposals in 3GPP standardization working groups include: the transmission of replicas within mini-slots (to reduce the latency) [18], the possibility for transmitting replicas across the slot border, or the concept of periodicity boundary [19]. These studies propose interesting variants of the *K*-repetitions scheme. However, it is yet unknown whether 5G NR grant-free scheduling with *K*-repetitions and shared resources can really support URLLC communications with strict reliability and latency requirements under the presence of aperiodic or sporadic traffic. This traffic is critical in many verticals, for example in Industry 4.0. In this context, this study conducts an in-depth evaluation of 5G NR grant-free scheduling with *K*-repetitions and shared resources in the presence of aperiodic or sporadic traffic. The study identifies the reliability and latency levels that can be achieved with 5G NR grant-free scheduling, and identifies its current limitations. The study analyzes the impact of the number of UEs in the network, the number of reserved radio resources, and the number of replicas *K*. The study also analyzes for the first time the impact of self-collisions. The conducted analysis helps to identify the reliability and latency levels that can be achieved based on network deployments and configuration options for 5G NR grant-free scheduling.

It should be noted that 3GPP standards define the possibility of utilizing grant-free scheduling and transmitting *K* replicas, but do not define a specific scheme to be implemented. This study is based on the implementation of 5G NR grant-free scheduling with *K* replicas and shared resources proposed in [15]. This implementation is chosen because it has been specifically designed to guarantee stringent URLLC latency and reliability requirements. To this aim, the implementation transmits original packets and all of the replicas using grant-free scheduling on shared radio resources. A different approach is proposed in [13] where dedicated resources are used to transmit the original packets, and shared resources are used for the following replicas. This approach can increase the delay compared to [15] if grant-based scheduling is utilized to allocate the dedicated resources. The efficient utilization of resources could also be compromised if dedicated resources were reserved for each UE when supporting applications with aperiodic traffic. The implementation of 5G NR grant-free scheduling with *K*–repetitions and shared resources proposed in [15] is therefore better suited to support URLLC applications with aperiodic or sporadic traffic.

## 3. Grant-Free Scheduling

**Figure 1:**
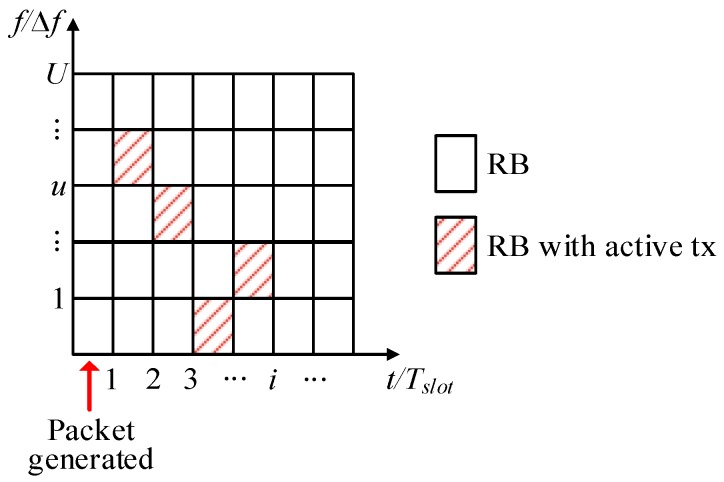
Illustration of the Fifth Generation New Radio (5G NR) resource grid map: Transmission of a data packet with four repetitions and a random selection of Resource Blocks (RBs) per slot.

This paper uses grant-free scheduling with *K*-repetitions and shared resources to evaluate the reliability and latency levels that can be achieved in the presence of aperiodic traffic. Following [20], reliability for URLLC services is defined as the percentage of data packets that are successfully delivered before the latency deadline *L* established by the service or application. Following 3GPP standards [11], UEs transmit the same data packet in *K* consecutive transmission slots with a duration $T_{slot}.$ The UE randomly selects an RB (Resource Block) for each transmission from the *U* RBs available per $T_{slot}.$ This is illustrated in Figure 1 that represents the time/frequency resource grid map in 5G NR, where the unit is an RB. In 5G NR, a wideband channel is divided into sub-frames, slots and RBs. An RB is the smallest unit of frequency resources that can be allocated to a UE. Without loss of generality, this study considers a numerology *µ* equal to 3 with a subcarrier spacing of 120 kHz [21]. An RB is then 1440 kHz (∆*f*) wide in frequency (12 sub-carriers of 120 kHz) and lasts for one time slot with the duration $T_{slot}$ equal to 0.125 ms.

The reliability at the medium access level that can be achieved with grant-free scheduling with *K*-repetitions and shared resources depends upon two main factors. The first factor is the possibility that a packet is not correctly received due to the collision of all its *K* replicas with other transmissions; this is due to the random selection of the RB for the transmission of each replica. The study in [15] showed that the possibility to successfully deliver a packet increases with the number *K* of replicas. The second factor is the effect of self-collisions. A self-collision occurs when a UE has to transmit a new packet, and the transmission of the *K* replicas of the previous packet has not finished. If this happens, the new packet must be stored, and its transmission is delayed until all the replicas of the previous packet have been transmitted. This delay can result in the case that the new packet cannot be delivered within the latency limit, and hence self-collisions can impact the reliability of URLLC services. It is important then that the reliability (or probability that a packet is correctly received before the latency deadline) of grant-free scheduling with *K*-repetitions and shared radio resources is computed considering both the effect of collisions from other UEs, and the effect of self-collisions. In this case, the reliability or probability $P_{rel}$ that a packet is correctly received by the BS must consider the probability $P_{sc}$ that the transmission of the *K* replicas of a packet is not completed before the latency deadline *L* due to the effect of self-collisions. For the packets that are not affected by the effect of self-collisions, it must be considered the probability $P_{c}$ that a packet is not correctly received due to the collision of all its *K* replicas with other transmissions. Hence, $P_{rel}$ can be expressed as:

(1) $$P_{rel}=1-\left(P_{sc}+(1-P_{sc})\cdot P_{c}\right)$$

In [15], its authors presented an expression to approximate the probability $P_{c}$ of the collision of the *K* replicas of a packet with the transmission of other UEs. The expression was derived in scenarios where *N* UEs share the same pool of RBs. However, [15] did not analyze the impact of self-collisions, since the study only considered low values of *K* (equal to or lower than 4). For these low values, self-collisions might not have an impact upon the reliability, as will be later shown. In this paper, we analytically derive the exact probability of any collision of the *K* replicas of a packet with packets transmitted by other UEs ($P_{c}$). We also quantify the impact of self-collisions ($P_{sc}$), and analytically compute the reliability that can be achieved by grant-free scheduling with *K*-repetitions and shared resources ($P_{rel}$). These analytical expressions are a valuable contribution to the community since they can be easily utilized to evaluate 5G NR grant-free scheduling. The availability of these exact analytical expressions is particularly useful when considering applications with very demanding reliability and latency URLLC requirements. This is the case of certain Industry 4.0 applications. For example, motion control requires a maximum latency of 1 ms and a reliability of 1–10^−6^. Control-to-control applications require a maximum latency of 4 ms and a reliability of 1–10^−8^. Factory automation applications usually demand maximum latency values in the range 0.25–2.5 ms and reliability levels up to 1–10^−9^. In this case, simulations can be very computationally expensive if we want to compute the packet reception rate (1 − $P_{c}$) with reliability demands in the order of 1–10^−6^ to 1–10^−9^. In these scenarios, errors are very rare, and we need long and computationally expensive simulations to achieve accurate results. The analytical methodology utilized in this study is then an adequate and efficient tool for scenarios with demanding URLLC communication requirements.

### 3.1. Collisions with Other UEs

First, we focus on the probability $P_{c}$ that a packet is not correctly received due to the collisions of its *K* replicas with the packets transmitted by other UEs. To this end, we consider UL transmissions and *N* UEs within a single cell with aperiodic traffic. Packets are generated by each UE following a Poisson distribution with exponential inter-arrival time. The average packet inter-arrival time is equal to 1/*λ*, where *λ* is the average number of packets generated per second. We consider the transmission of small packets with a size of 32 bytes [22], and we assume without loss of generality that each packet requires only one RB.

The probability $P_{g}$ that one or more packets are generated for a UE in a time period $T_{slot}$ is equal to:

(2) $$P_{g}=1-\exp\left(-T_{slot}\cdot \lambda \right)$$

We define $R_{i}$ as the set of UEs for which a new packet could be generated in a slot $s_{i}$ (the slot has a time duration equal to $T_{slot}$). Here, $n_{i}$ is the number of UEs that do have a new packet to transmit in $s_{i}.$ This $n_{i}$ can then take any value between 0 and the cardinality of $R_{i}$. The probability $P_{tx}(n_{i},R_{i})$ that $n_{i}$ UEs from the set $R_{i}$ of UEs have new packets to be transmitted in $s_{i}$ with duration $T_{slot}$ is equal to:

(3) $$P_{tx}(n_{i},R_{i})=\left(\begin{array}{c} \left|R_{i}\right|\\ n_{i} \end{array}\right)\cdot P_{g}{}^{n_{i}}\cdot {\left(1-P_{g}\right)}^{\left|R_{i}\right|-n_{i}}$$

where $\left|R_{i}\right|$ represents the number of elements or the cardinality of the set $R_{i}.$

A packet will not be successfully delivered to the BS if all its *K* replicas collide with the trans-missions of other UEs. A UE has an active transmission in $s_{i}$ if it generated a new data packet in the previous slots $s_{i-(K-1)},$ …, $s_{i-1},$ and $s_{i}$. If this is the case, then the UE would be transmitting one of the *K* replicas in $s_{i}$. We denote as $n_{i}^{act}$ the number of UEs with active transmissions in $s_{i}$. The probability $P_{nrc}(n_{i}^{act},U)$ that $n_{i}^{act}$ UEs do not collide with a given UE is equal to the probability that they do not select the same RB at a given slot for their next transmission as the UE under study. $P_{nrc}(n_{i}^{act},U)$ is given by:

(4) $$P_{nrc}(n_{i}^{act},U)={\left(\begin{array}{c} U-1\\ U \end{array}\right)}^{n_{i}^{act}}$$

Equations (2)–(4) are necessary to compute the probability $P_{c}$ that a packet is not correctly received at the BS due to the collision of all its *K* replicas with the transmissions of other UEs. To compute $P_{c}$, let us consider the case of a particular UE_1_ that has to transmit the *K* replicas of a packet in slots $s_{i}$, $s_{i+1}$, …, $s_{i+K-1}$. For the sake of clarity, we consider an example with *K* = 4, and $s_{i}$ corresponding to $s_{3}$. $P_{c}$ is then equal to the probability of collision of the 4 replicas transmitted in $s_{3},$ $s_{4}$, $s_{5}$, and $s_{6}$, which is represented by $P_{rc}\left(s_{3},s_{4},s_{5},s_{6}\right)$:

(5) $$P_{c}=P_{rc}\left(s_{3},s_{4},s_{5},s_{6}\right)$$

To determine $P_{rc}\left(s_{3},s_{4},s_{5},s_{6}\right)$, we first study the probability $P_{rc}\left(s_{3}\right)$ that the replica of the packet transmitted in $s_{3}$ collides with a transmission from any other UE. $P_{rc}\left(s_{3}\right)$ is given by the probability that one or more UEs (in addition to UE_1_) have an active transmission in $s_{3}$ (i.e., $n_{3}^{act}\geq$ 1), and that one or more of the $n_{3}^{act}$ UEs select the same RB as UE_1_ for their transmission. $n_{3}^{act}$ is equal to *n*_0_ + *n*_1_ + *n*_2_ + *n*_3_, and the probability $P_{rc}\left(s_{3}\right)$ has to consider all possible combinations of *n*_0_, *n*_1_, *n*_2_ and *n*_3_ that result in $n_{3}^{act}\geq$ 1. The probability $P(n_{3}^{act}\geq 1)$ can then be expressed as:

(6) $$P(n_{3}^{act}\geq 1)=\sum_{n_{0}=n_{0}^{min}}^{n_{0}^{max}}\left\{P_{tx}(n_{0},R_{0})\cdot \sum_{n_{1}=n_{1}^{min}}^{n_{1}^{max}}\langle P_{tx}(n_{1},R_{1})\cdot \sum_{n_{2}=n_{2}^{min}}^{n_{2}^{max}}\left[P_{tx}(n_{2},R_{2})\cdot \sum_{n_{3}=n_{3}^{min}}^{n_{3}^{max}}P_{tx}(n_{3},R_{3})\right]\rangle \right\}$$

where $n_{i}^{max}$ and $n_{i}^{min}$ represent the maximum and minimum possible values of *n_i_* in each slot, and are equal to:

(7) $$n_{i}^{max}=\left|R_{i}\right|,\;\forall i\leq 3$$

(8) $$n_{i}^{min}=\{\begin{array}{ll} 1 & \mathrm{if}\;i=3\;\&\;\left|R_{i}\right|=N-1\\ 0 & \mathrm{otherwise} \end{array},\;i\leq 3$$

where $R_{i}$ is the set of UEs that could have a new packet to be transmitted in $s_{i}.$ $R_{i}$ is equal to the total number of UEs (*N*) minus UE_1_ and all active UEs in the slot previous to $s_{i}.$ The cardinality of $R_{i}$ is then equal to:

(9) $$\left|R_{i}\right|=N-1-\sum_{j=\max\{i-3,0\}}^{i-1}n_{j},\;i\leq 3$$

It should be noted that $n_{i}^{min}$ is equal to 0 or 1 in order to guarantee that $n_{3}^{act}$ is equal to or higher than one. $n_{i}^{act}$ can be expressed as:

(10) $$n_{i}^{act}=\sum_{j=\max\{i-3,0\}}^{i}n_{j},\;i\leq 3$$

To achieve finally the expression of $P_{rc}\left(s_{3}\right)$, we need to incorporate to the expression of $P(n_{3}^{act}\geq 1)$ in (6) the probability that one or more of the $n_{3}^{act}$ UEs select the same RB as UE_1_ for their transmissions. This probability is equal to 1 − $P_{nrc}(n_{3}^{act},U)$. $P_{rc}\left(s_{3}\right)$ is then calculated as:

(11) $$P_{rc}(s_{3})=\sum_{n_{0}=n_{0}^{min}}^{n_{0}^{max}}\left\{P_{tx}(n_{0},R_{0})\cdot \sum_{n_{1}=n_{1}^{min}}^{n_{1}^{max}}\langle P_{tx}(n_{1},R_{1})\cdot \sum_{n_{2}=n_{2}^{min}}^{n_{2}^{max}}\left[P_{tx}(n_{2},R_{2})\cdot \sum_{n_{3}=n_{3}^{min}}^{n_{3}^{max}}\left\{P_{tx}(n_{3},R_{3})\cdot (1-P_{nrc}(n_{3}^{act},U))\right\}\right]\rangle \right\}$$

The probability of collision of the replica transmitted in $s_{4}$ depends upon the number $n_{4}^{act}$ of UEs with active transmissions in $s_{4}$. This $n_{4}^{act}$ depends on the number *n*_1_, *n*_2_, *n*_3_ and *n*_4_ of UEs that have new packets to transmit in $s_{1}$, $s_{2}$, $s_{3}$, and $s_{4}$, respectively. The probability that UEs have new packets to transmit in $s_{1}$, $s_{2}$, and $s_{3}$ is already included in (11) $(P_{tx}(n_{1},R_{1}),$ $P_{tx}(n_{2},R_{2}),$ and $P_{tx}(n_{3},R_{3})$ respectively). In this context, $P_{rc}(s_{3})$ and $P_{rc}(s_{4})$ are not independent, and they must be calculated jointly. We then compute the joint probability $P_{rc}(s_{3},s_{4})$ that the replicas transmitted in $s_{3}$ and $s_{4}$ collide with transmissions from other UEs. Computing $P_{rc}(s_{3},s_{4})$ only requires including in (11) the probability that there are UEs with new packets to be transmitted in $s_{4}$ (i.e., $P_{tx}(n_{4},R_{4})),$ and the probability that one or more of the active $n_{4}^{act}$ UEs in $s_{4}$ select the same RB for their transmission than UE_1_. $P_{rc}(s_{3},s_{4})$ can then be expressed as:

(12) $$\begin{array}{l} P_{rc}(s_{3},s_{4})=\sum_{n_{0}=n_{0}^{min}}^{n_{0}^{max}}\{P_{tx}(n_{0},R_{0})\cdot \sum_{n_{1}=n_{1}^{min}}^{n_{1}^{max}}\langle P_{tx}(n_{1},R_{1})\cdot \sum_{n_{2}=n_{2}^{min}}^{n_{2}^{max}}[P_{tx}(n_{2},R_{2})\cdot \\ \sum_{n_{3}=n_{3}^{min}}^{n_{3}^{max}}\langle \left\{P_{tx}(n_{3},R_{3})\cdot (1-P_{nrc}(n_{3}^{act},U))\right\}\cdot \sum_{n_{4}=n_{4}^{min}}^{n_{4}^{max}}\left\{P_{tx}(n_{4},R_{4})\cdot (1-P_{nrc}(n_{4}^{act},U))\right\}\rangle ]\rangle \} \end{array}$$

where $n_{4}^{act}$, $\left|R_{4}\right|,$ $n_{4}^{max}$ and $n_{4}^{min}$ are defined as:

(13) $$n_{4}^{act}=\sum_{j=1}^{4}n_{j}$$

(14) $$\left|R_{4}\right|=N-1-\sum_{j=1}^{3}n_{j}$$

(15) $$n_{4}^{max}=\left|R_{4}\right|$$

(16) $$n_{4}^{min}=\{\begin{array}{ll} 1 & \mathrm{if}\;\left|R_{4}\right|=N-1\\ 0 & \mathrm{otherwise} \end{array}$$

The process followed to account for possible collisions of the replicas transmitted in $s_{5}$ and $s_{6}$ is similar to that considered for $s_{4}$. $P_{c}$ can then be expressed as follows when *K* = 4:

(17) $$\begin{array}{l} P_{c}=\sum_{n_{0}=n_{0}^{min}}^{n_{0}^{max}}\{P_{tx}(n_{0},R_{0})\cdot \sum_{n_{1}=n_{1}^{min}}^{n_{1}^{max}}\langle P_{tx}(n_{1},R_{1})\cdot \sum_{n_{2}=n_{2}^{min}}^{n_{2}^{max}}[P_{tx}(n_{2},R_{2})\cdot \\ \sum_{n_{3}=n_{3}^{min}}^{n_{3}^{max}}\langle \left\{P_{tx}(n_{3},R_{3})\cdot (1-P_{nrc}(n_{3}^{act},U))\right\}\cdot \sum_{n_{4}=n_{4}^{min}}^{n_{4}^{max}}[\left\{P_{tx}(n_{4},R_{4})\cdot (1-P_{nrc}(n_{4}^{act},U))\right\}\cdot \\ \sum_{n_{5}=n_{5}^{min}}^{n_{5}^{max}}\langle \left\{P_{tx}(n_{5},R_{5})\cdot (1-P_{nrc}(n_{5}^{act},U))\right\}\cdot \sum_{n_{6}=n_{6}^{min}}^{n_{6}^{max}}\left\{P_{tx}(n_{6},R_{6})\cdot (1-P_{nrc}(n_{6}^{act},U))\right\}\rangle ]\rangle ]\rangle \} \end{array}$$

where $n_{i}^{act}$, $\left|R_{i}\right|,$ $n_{i}^{max}$ and $n_{i}^{min}$ ∀*i* ∊ [0, 2⋅*K*−1] are defined as:

(18) $$n_{i}^{act}=\sum_{j=\max\{i-(K-1),0\}}^{i}n_{j}$$

(19) $$\left|R_{i}\right|=N-1-\sum_{j=\max\{i-(K-1),0\}}^{i-1}n_{j}$$

(20) $$n_{i}^{max}=\left|R_{i}\right|$$

(21) $$n_{i}^{\min}=\{\begin{array}{ll} 1 & \mathrm{if}\;i\geq K-1\;\&\;\;\left|R_{i}\right|=N-1\\ 0 & \mathrm{otherwise} \end{array}$$

The process illustrated for *K* = 4 can be followed to compute $P_{c}$ for any value of *K*. As shown in (22), $P_{c}$ can be computed using the auxiliary function $h_{i}(K,N,U)$ defined in (23) with *i* equal to cero. To simplify the notation, $h_{i}(K,N,U)$ is also represented as $h_{i}$ in (22) and (23). As it can be observed in (23), $h_{0}$ depends on $h_{1}$, and in general, $h_{i}$ depends on $h_{i+1}$, until $h_{2K-1}$.

(22) $$P_{c}(K,N,U)=h_{0}(K,N,U)=h_{0}$$

(23) $$h_{i}=\{\begin{array}{ll} \sum_{n_{i}=n_{i}^{min}}^{n_{i}^{max}}\left[P_{tx}(n_{i},R_{i})\cdot h_{i+1}\right] & \mathrm{if}\;i\in [0,K)\\ \sum_{n_{i}=n_{i}^{min}}^{n_{i}^{max}}\left[P_{tx}(n_{i},R_{i})\cdot (1-P_{nrc}(n_{5}^{act},U))\cdot h_{i+1}\right] & \mathrm{if}\;i\in [K,\;2\cdot K-1)\\ \sum_{n_{i}=n_{i}^{min}}^{n_{i}^{max}}\left[P_{tx}(n_{i},R_{i})\cdot (1-P_{nrc}(n_{5}^{act},U))\right] & \mathrm{if}\;i=2\cdot K-1 \end{array}$$

The parameters $n_{i}^{act}$, $\left|R_{i}\right|,$ $n_{i}^{max}$ and $n_{i}^{min}$ in (23) correspond to those expressed in (18)–(21).

### 3.2. Self-Collisions

**Figure 2:**
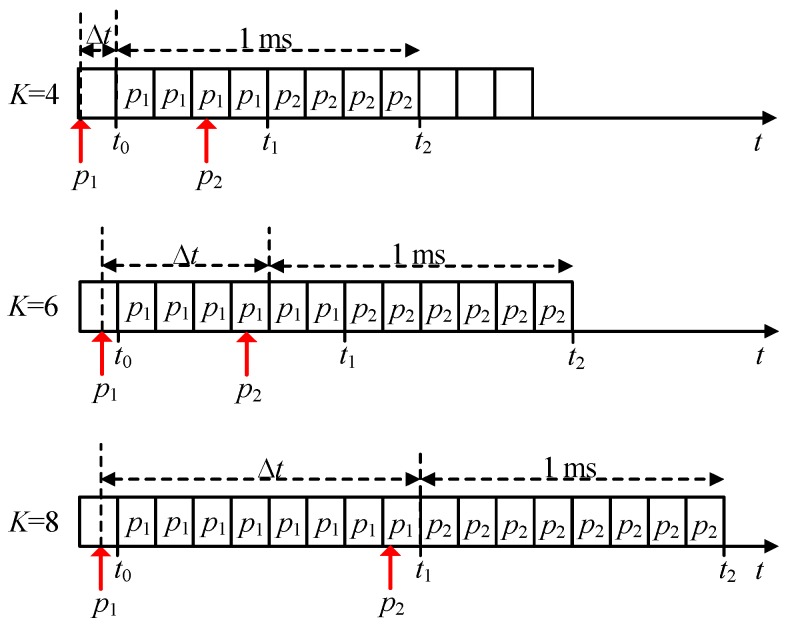
Scenarios with possible self-collisions (*L* = 1 ms and *K* = 4, 6 and 8).

The effect of self-collisions is illustrated in Figure 2. We may suppose that a UE starts transmitting a packet *p*_1_ that was generated before *t*_0_. Let us then suppose then that a second packet *p*_2_ is generated before the *K* replicas of the previous packet *p*_1_ have been transmitted. This is a self-collision. If a self-collision happens, *p*_2_ can be stored, and its transmission will start after the UE has transmitted the *K*^th^ replica of *p*_1_ (i.e., at *t*_1_ in Figure 2). The transmission of the *K* replicas of *p*_2_ will finish at *t*_2_ that is equal to:

(24) $$t_{2}=2\cdot K\cdot T_{slot}+t_{0}$$

The transmission of the *K* replicas of *p*_2_ may finish after the latency deadline *L*, due to the time *p*_2_ being stored as the *K* replicas of *p*_1_ are being transmitted. We then analyze the probability $P_{sc}$ that the transmission of *K* replicas of a packet is not completed before *L* due to the effect of self-collisions. This probability depends upon the number of replicas *K* and on the time instant at which *p*_2_ was generated. Figure 2 illustrates how self-collisions affect the probability of completing the transmission of *p*_2_ before *L*, with *L* equal to 1 ms. *L* = 1 ms implies that the maximum number of replicas *K* that can be transmitted per packet is 8. However, it is possible to transmit less than 8 replicas, and Figure 2 represents the case in which *K* is set equal to 4, 6 or 8. *p*_2_ can be transmitted before the deadline *L* if it is generated at any time instant after *t*_2_ − *L*, where *t*_2_ is the time at which the transmission of the *K* replicas of *p*_2_ is finished (the transmission of *p*_2_ starts when the transmission of the *K* replicas of *p*_1_ has finished at *t*_1_). If *p*_2_ is generated before *t*_2_ − *L*, it is not possible to complete the transmission of the *K* replicas of *p*_2_ before the latency deadline *L*. $P_{sc}$ can then be computed as the probability that the time between the generation of two consecutive packets at a UE falls within the interval [0, ∆*t*], where ∆*t* represents the time difference between *t*_2_ − *L* and the time $t_{p_{1}}$ at which *p*_1_ is generated (see (26)). $P_{sc}$ can then be expressed as:

(25) $$P_{sc}(\Delta t)=\int_{0}^{\Delta t}\lambda \cdot e^{-t\cdot \lambda }\cdot dt$$

(26) $$\Delta t=t_{2}-L-t_{p_{1}}=2\cdot K\cdot T_{slot}-L-t_{p_{1}}$$

As shown in (25) and (26), the negative effect of self-collisions increases with the value of *K*, since *K* influences the time a packet might be stored until the transmission of the previous packet is finished. However, increasing the number *K* of replicas transmitted for each packet is preferred, in order to combat possible collisions with other UEs sharing the same pool of radio resources. The next section will analyze both the effect of collisions from other UEs and the effect of self-collisions to analyze the reliability achievable with the grant-free scheduling with *K*-repetitions and shared radio resources.

## 4. Validation

This section validates the analytical expressions derived in Section 3.1 to calculate the probability $P_{c}$ that a packet is not correctly received due to packet collisions with other UEs. To this aim, we compare the results achieved with the analytical expressions, with that obtained through simulations.

We have implemented a system level simulator in Matlab™ that accurately models the 5G NR grant-free scheduling process with *K*-repetitions and shared resources. The simulator emulates a single cell with *N* UEs that generate aperiodic traffic. Each UE models the packet traffic arrival, using a Poisson distribution with exponential inter-arrival time. The average packet inter-arrival time is equal to *1/λ*, where *λ* is the average number of packets generated per second. The simulator implements the time/frequency resource grid map of 5G NR. The time and frequency duration of RBs is configurable based on the considered 5G NR numerology *µ*. It is possible to also configure the number *U* of RBs available per time slot. The number *K* of replicas can also be configured in the simulation platform.

**Figure 3:**
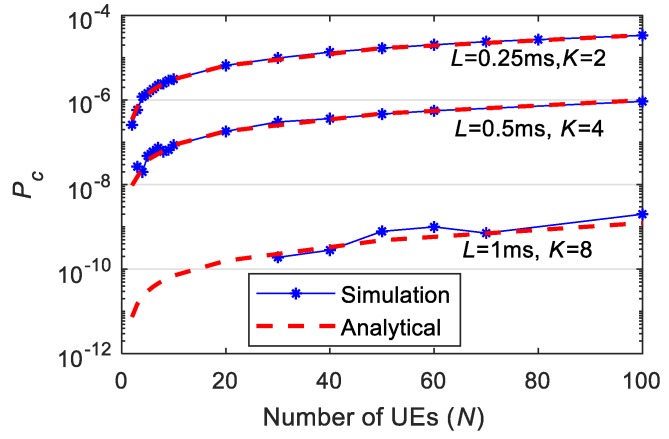
Comparison of analytical and simulation results for different latency requirements *L* and number of repetitions *K* (*U* = 6, *λ* = 0.1 packets).

We have conducted a large number of simulations to ensure the accuracy of the simulation results, and compare them to those obtained with our analytical expressions and methodology. Simulations are here shown for *K* equal to 2, 4 and 8, *λ* equal to 0.1 packets, *µ* equal to 3, and *U* equal to 6 RBs per slot. UEs transmit small packets with a size of 32 bytes [22] that can be transmitted in a single RB. Figure 3 compares the value of $P_{c}$ achieved analytically and through simulations for a varying number *N* of users in the cell. The figure shows that the results achieved analytically precisely match those obtained through the simulations. Similar trends have been observed for other values of the parameters. The results achieved clearly validate the proposed methodology and the analytical expressions presented in Section 3.1.

It is important to highlight that this study focuses on URLLC applications that demand very high reliability levels. In simulations, we compute the number of packets for which the *K* replicas have collided with those packets transmitted by other UEs, and then compute the achieved reliability ($P_{rel}=1-P_{c}$). It is rare that all *K* replicas of a packet collide with transmissions from other UEs for low values of *N*. This is particularly the case when *K* increases. In this context, the computational cost of simulations significantly increases if we want to achieve accurate statistical results. This explains why simulation results are not shown for values of *N* below 30 when *K* = 8. It also highlights the value of our analytical expressions and methodology to estimate the performance of 5G NR grant-free scheduling for demanding URLLC applications and aperiodic traffic.

## 5. Performance Evaluation

This section evaluates the capacity of 5G NR grant-free scheduling with *K*-repetitions and shared resources to meet the reliability and latency requirements of URLLC services. To this aim, we use the analytical expressions that are derived in Section 3 and were validated in the previous section. Reliability for URLLC services is defined as the percentage $P_{rel}$ of data packets that are successfully received by the BS before the latency deadline established by the service or application. In this study, we analyze first the reliability, considering only the effect of collisions from other UEs. This study analyzes then the impact of self-collisions on the capacity of 5G NR grant-free scheduling with *K-*repetitions and shared resources to achieve the reliability levels demanded by URLLC services. This is particularly relevant, as this study extends the state of the art by evaluating the capacity of 5G NR grant-free scheduling to sustain reliability levels even higher than 1–10^−9^. This study also evaluates the performance of 5G NR grant-free scheduling as a function of the number of UEs, the number of reserved radio resources, and the number *K* of replicas.

**Figure 4:**
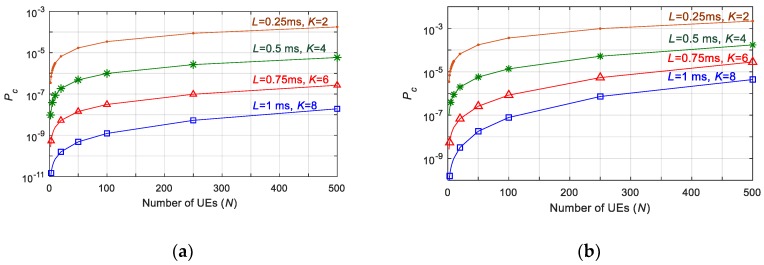
$P_{c}$ as function of the number of User Equipments (UEs) and for different latency requirements *L*: (**a**) *λ* = 0.1 packets; (**b**) *λ* = 1 packet.

The performance of 5G NR grant-free scheduling is evaluated considering a single cell with *N* UEs. Packets are generated by each UE following a Poisson process with exponentially inter-arrival time. The average packet inter-arrival time is equal to 1/*λ*, where *λ* is the average number of packets generated per second. UEs transmit small packets with a size of 32 bytes [22]. Radio resources are divided in 6 × 12 subcarriers (i.e., *U* = 6) with a subcarrier spacing of 120 kHz (i.e., $T_{slot}$ = 0.125 ms). Figure 4 shows the probability $P_{c}$ that a packet is not correctly received at the BS due to the collisions from other UEs experienced by all of the replicas of a packet (This would correspond to the reliability achieved with 5G NR grant-free scheduling if there were no self-collisions, i.e., *P_sc_* = 0 and *P_rel_* = 1 − *P_c_*). The figure shows the value of $P_{c}$ that can be achieved as a function of the number of UEs for latency requirements (*L*) of 0.25, 0.5, 0.75 and 1 ms. We focus on services with the most stringent latency requirements, given the challenge to satisfy high reliability levels when latency decreases [23]. For each value of *L*, the grant-free scheduling scheme is executed with the maximum possible number of replicas *K* that can be transmitted within the required latency. For example, if the maximum latency *L* that can be tolerated is equal to 1 ms, the maximum number of replicas *K* that can be transmitted within 1 ms is equal to 8 (*L* = 1 ms corresponds to 8*⋅*$T_{slot}$ when $T_{slot}$ = 0.125 ms). Figure 4 also shows the performance achieved for two values of *λ* (0.1 and 1 packet(s)). The results depicted in Figure 4 clearly show that reducing the probability $P_{c}$ of not receiving a packet to values as low as 10^−9^, (and hence reaching reliability levels of 1–10^−9^ when the effect of self-collisions is not considered), can only be achieved with high values of *K* and values of *L* equal to 0.75 or 1 ms. Figure 4 also shows that the probability $P_{c}$ increases with the number of UEs, since the risk of collision is higher. As a result, the capacity of 5G NR grant-free scheduling to support high reliability levels is significantly decreased as the number of UEs to be supported increases. Figure 4 also shows that the difficulty in supporting high reliability levels increases with *λ*, since the probability $P_{c}$ increases as a result of a higher risk of collision between UEs.

**Figure 5:**
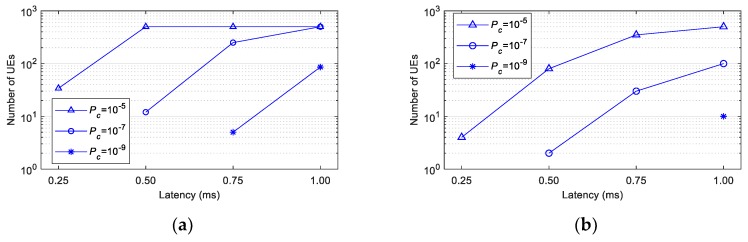
Number of UEs supported with different requirements (*L* and *P_rel_* = 1 − *P_c_*, when *P_sc_* = 0): (**a**) *λ* = 0.1 packets; (**b**) *λ* = 1 packet.

Figure 5 depicts the number of UEs that can be supported with a given latency requirement (*L*) and a reliability of $P_{rel}=1-P_{c}$ when $P_{sc}=$ 0. It is important to remember that *L* establishes the maximum number of replicas *K* that can be transmitted. The results (the number of supported UEs) for each value of *L* in Figure 5 have been obtained for the maximum value of *K* permitted by *L* (*K* equal to 2, 4, 6 and 8 for *L* equal to 0.25, 0.5, 0.75 and 1 ms, respectively). The Release 15 of the 3GPP standards [22] establishes URLLC requirements with a latency of *L* = 1 ms and a reliability target of 1–10^−5^. Figure 5 shows that grant-free scheduling with *K*-repetitions and shared resources can achieve a reliability equal to 1–10^−5^ with only *K* = 2 if we do not consider self-collisions. Grant-free scheduling with *K* = 2 can also guarantee a latency as low as 0.25 ms. For low values of the packet generation rate (i.e., *λ =* 0.1 packets), grant-free scheduling with 2 repetitions can support up to 34 UEs with a reliability of 1–10^−5^ and *L* = 0.25 ms if we do not consider self-collisions. The number of UEs that can be supported decreases with *λ*, since the risk of collision with other UEs increases when each UE transmits more packets per second. For example, only 4 UEs can be supported with *L* = 0.25 ms and a reliability of 1–10^−5^ when *λ* = 1 packet. If the latency requirement is relaxed to 0.5 ms or even higher, grant-free scheduling can support more than 500 UEs with only *K* = 4 when *λ =* 0.1 packets. If *λ* increases, grant-free scheduling can only guarantee the required reliability for 500 UEs if the latency requirement is 1 ms, and each UE can transmit 8 replicas of the same packet. These results show that the reliability and latency levels that can be achieved with grant-free scheduling depend upon configuration parameters (e.g., *K*), the traffic (e.g., *λ*) and the number of UEs supported. An adequate configuration and optimization of grant-free scheduling based on the network conditions could help support stringent reliability and latency levels. However, it is important to note that these results are achieved without considering self-collisions. The impact of self-collisions might be non-negligible when, for example, *K* and/or *λ* increase.

The Release 16 of 3GPP standards for 5G NR [2] defines use cases with higher reliability requirements (up to 1–10^−6^). Some Industry 4.0 applications (e.g., factory automation) require even higher reliability levels (up to 1–10^−9^), as discussed in [3]. It is then important analyzing whether grant-free scheduling with *K*-repetitions and shared resources can guarantee reliability levels of the order of 1–10^−9^. Figure 4 and Figure 5 show that grant-free scheduling can only guarantee very high reliability levels with high values of *K*, which limits the latency requirements (*L*) that can be satisfied. For example, a probability to correctly receive a packet equal to 1–10^−7^ cannot be guaranteed when *L* < 0.5 ms, even for the lower packet generation rates. If the reliability requirement increases to $P_{rel}=$ 1–10^−9^, grant-free scheduling can only support 5 UEs with *L* = 0.75 ms and *λ* = 0.1 packets. It can support 86 UEs if the latency requirement is relaxed to 1 ms. However, if *λ* increases to 1 packet then grant-free scheduling can only support 10 UEs with a reliability of 1–10^−9^ even if *L* is equal to 1 ms.

**Figure 6:**
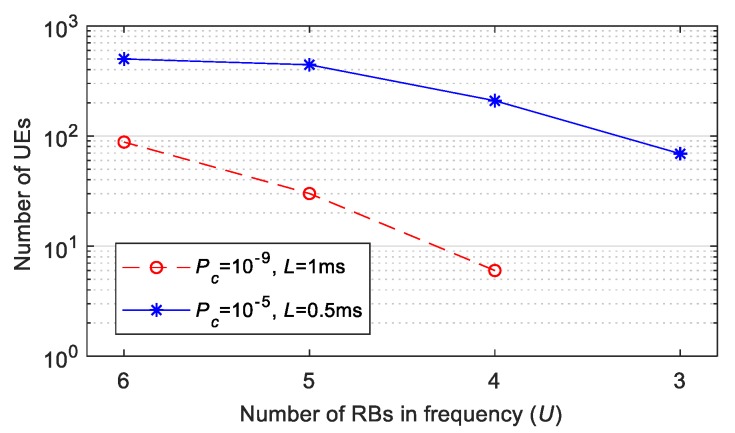
Number of UEs supported for a given *L* and *P_rel_* = 1 − *P_c_* with *P_sc_* = 0 as a function of the number *U* of available RBs per T_slot_ (*λ* = 0.1 packets).

Figure 6 shows the impact of *U* upon the performance of the grant-free scheduling scheme with *K*-repetitions and shared resources. *U* is the number of available RBs (Resource Blocks) per $T_{slot}$. In particular, Figure 6 depicts the number of UEs that can be supported with a given reliability and latency *L* when *U* decreases and *λ* is set equal to 0.1 packets (the reliability is equal to *P_rel_* = 1 − *P_c_* when the effect of self-collisions is not taken into account, i.e., *P_sc_* = 0). Figure 6 shows that the number of UEs that grant-free scheduling with *K*-repetitions can support for a given set of requirements strongly depends upon the number of RBs available. UEs randomly select an RB for each transmission from the *U* RBs available per slot. The probability that several UEs select the same RB for their transmissions increases when the number of RBs per slot decreases. Consequently, the probability $P_{c}$ that a packet is not correctly received due to packet collisions, increases. In addition, the number of UEs that can achieve a target reliability level also decreases when the number of RBs per slot decreases. For example, 443 UEs can be supported with *L* = 0.5 ms (and hence *K* = 4) and $P_{c}$ = 10^−5^ when *U* is equal to 5 RBs. This number decreases to 69 UEs when *U* decreases to 3 RBs. This is a significant reduction of 84%. This reduction increases when the reliability demand increases. For example, 86 UEs can be supported with $P_{c}$ = 10^−9^ and *L* = 1 ms (and hence *K* = 8) when *U* is equal to 6. However, only 6 UEs can achieve these values of $P_{c}$ and *L* if *U* decreases to 4 (i.e., a 93% reduction).

All previous results have been derived without considering the effect of self-collisions. Self-collisions were illustrated in Figure 2, and the probability of self-collision was derived in Section 3.2. As previously described, if a packet *p*_2_ is generated before the *K* replicas of the previous packet *p*_1_ have been transmitted, *p*_2_ will be stored and transmitted after completing the transmission of the *K* replicas of *p*_1_. Due to the time that *p*_2_ is stored, the transmission of its *K* replicas may finish after the latency deadline *L*. As presented in Section 3.2, it is not possible to complete the transmission of the *K* replicas of *p*_2_ before the latency deadline *L* if *p*_2_ is generated before *t*_2_ − *L* (*t*_2_ is the time at which the transmission of the *K* replicas of *p*_2_ is finished as shown in Figure 2). This results in that the probability $P_{sc}$ (the probability that the transmission of *K* replicas of a packet is not completed before *L* due to the effect of self-collisions) is equal to the probability that the time between the generation of two consecutive packets at a UE falls within the interval [0, ∆*t*], where ∆*t* represents the time difference between *t*_2_ − *L* and the time $t_{p_{1}}$ at which *p*_1_ is generated (see (25) and (26)).

We consider that packets are generated following a Poisson process with exponential inter-arrival time. As a result, ∆*t* is homogeneously distributed between ∆*t*_1_ and ∆*t*_2_. For *K* = 4 in Figure 2, ∆*t*_1_ is equal to 0 and ∆*t*_2_ is equal to $T_{slot}$, since *p*_1_ can be homogeneously generated between *t*_0_ and *t*_0_ − $T_{slot}.$ When *K* = 6, ∆*t*_1_ is equal to 4 $\cdot T_{slot}$, and ∆*t*_2_ is equal to (4+1) $\cdot T_{slot}$, since *p*_1_ can be homogeneously generated between *t*_0_ and *t*_0_ − $T_{slot}.$ Similarly, ∆*t*_1_ and ∆*t*_2_ are equal to 8 $\cdot T_{slot}$ and (8+1) $\cdot T_{slot}$ for *K* = 8. Table 1 shows the value of $P_{sc}$ given in (26) when ∆*t* is equal to ∆*t*_1_ or ∆*t*_2_ considering *L* = 1 ms and *K* = 4, 6 and 8. ∆*t* = ∆*t*_1_ corresponds to the scenario where self-collisions are less probable, while ∆*t* = ∆*t*_2_ corresponds to the case in which they are more probable.

The results in Table 1 show that the probability of self-collision is non-negligible. For example, $P_{sc}$ can reach values equal to 1.25 × 10^−4^ and 9.99 × 10^−4^ when *K* is equal to 4 and 8, respectively, and *λ* = 1 packet. It is also important to highlight that a comparison of results in Figure 4 and Table 1 shows that $P_{sc}$ can be actually higher than $P_{c}.$ This is for example the case when *K* = 8: $P_{c}$ is lower than 10^−7^ and 10^−5^ for *λ* equal to 0.1 and 1 packet(s), respectively (Figure 4), while $P_{sc}$ is approximately equal to 10^−4^ and 10^−3^ (Table 1). Grant-free scheduling can hence be limited by the effect of self-collisions, in particular when *K* increases. It is then important that the reliability (or probability that a packet is correctly received before the latency deadline) of grant-free scheduling with *K*-repetitions and shared radio resources is computed considering both the effect of collisions from other UEs and the effect of self-collisions following (1).

**Figure 7:**
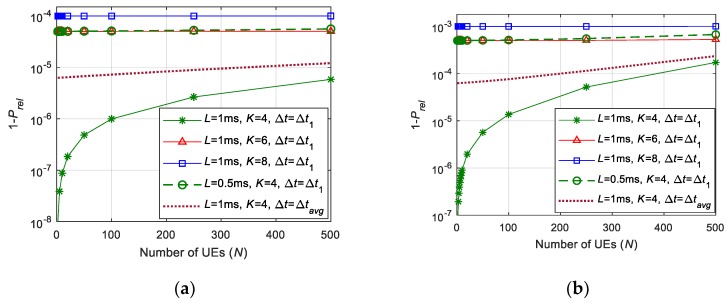
Reliability for different latency requirements *L* and number of repetitions *K* (*U* = 6): (**a**) *λ* = 0.1 packets; (**b**) *λ* = 1 packet.

Figure 7 plots 1 − $P_{rel}$ for different values of *K* and *L* when considering both $P_{c}$ and $P_{sc}$. The results are plotted considering ∆*t* = ∆*t*_1_ for computing $P_{sc}$. ∆*t* = ∆*t*_1_ corresponds to the case where self-collisions are less probable. Figure 4 shows that it is necessary to transmit a high number of replicas *K* within *L* to combat collisions from other UEs and correctly receive a packet at the BS. For example, Figure 4 shows that *K* must be equal to 8 in order to achieve $P_{rel}$ = 1–10^−9^ when $P_{sc}$ = 0 and *λ* is equal to 1 packet. However, Table 1 showed that the effect of self-collisions increases with *K* even to the point that self-collisions limit the reliability that can be achieved. This is actually shown in Figure 7 when we consider *L* = 1 ms. In principle, it could be possible to satisfy a 1 ms latency requirement if we transmit 4, 6 or 8 replicas of a packet. Figure 7 shows that if *K* = 4 and ∆*t* = ∆*t*_1_ (for computing $P_{sc}$ in (26)), the impact of self-collisions is not relevant, and the reliability levels of 1–10^−5^ can be satisfied for more than 500 UEs and 80 UEs when *λ* is equal to 0.1 and 1 packet(s), respectively; these results are in line with those observed in Figure 4 for *K* = 4. However, when *K* is equal to 6 or 8, the effect of self-collisions becomes more relevant (Table 1), and Figure 7 shows that it can actually limit the maximum reliability that can be achieved independently of the number of UEs. In fact, the maximum reliability that can be achieved is approximately equal to 1 − $P_{sc}$. In this case, for *K* = 8 and *λ* = 1 packet/s, the maximum reliability (when $P_{sc}$ is computed considering ∆*t* = ∆*t*_1_) that can be achieved is 1–10^−3^ when the latency requirement *L* is equal to 1 ms. It should be noted that reliability levels even higher than 1 − $P_{c}$ = 1–10^−9^ were achieved when the effect of self-collisions was not considered (Figure 4). The results discussed so far correspond to the scenario where $P_{sc}$ has been computed considering ∆*t* = ∆*t*_1_. This corresponds to the scenario where self-collisions are less probable. Figure 7 also shows the reliability that can be achieved with *L* = 1 ms and *K* = 4 when ∆*t* = ∆*t_avg_*. This ∆*t_avg_* is the average value of ∆*t*. ∆*t_avg_* = (∆*t*_1_ + ∆*t*_2_)/2, since ∆*t* is homogeneously distributed between ∆*t*_1_ and ∆*t*_2_. Figure 7 shows that in this case it is not possible to achieve a reliability higher than 1–6.3 × 10^−5^ and 1–6.3 × 10^−4^ when *λ* is equal to 0.1 and 1 packet(s). Figure 7 also shows that the reliability becomes again nearly independent of the number of UEs that are being supported. The degradation of reliability experienced from ∆*t* = ∆*t*_1_ to ∆*t* = ∆*t_avg_* is again due to a major relevance of the effect of self-collisions when we compute the reliability.

Expressions in (25) and (26) show that $P_{sc}$ also depends upon the latency requirement *L*. The effect of self-collisions is more relevant when the latency requirement is stricter. For example, Figure 7 shows that the effect of self-collisions already limits the maximum reliability that can be achieved when *K* = 4 if the latency requirement is equal to 0.5 ms. Latency requirements significantly influence the reliability levels that can be satisfied. This is the case because latency requirements limit the number *K* of replicas that can be sent for each packet. Figure 4 shows that the maximum reliability level that can be guaranteed depends on the latency requirements when only considering $P_{c}$. Figure 7 also shows that the effect of self-collisions becomes more relevant with stricter latency requirements. These results show that it is a challenge guaranteeing high reliability demands with very low latency levels.

**Figure 8:**
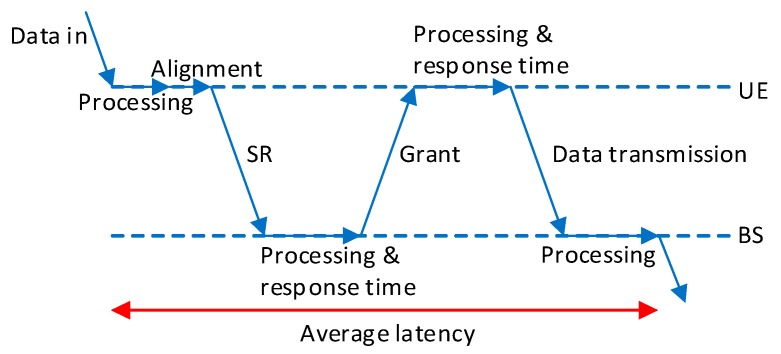
Latency introduced in grant-based scheduling.

The results in Figure 7 demonstrate that current 5G NR grant-free scheduling with *K*-repetitions and shared resources cannot guarantee some of the more demanding reliability and latency levels. However, it is important emphasizing that other proposals cannot meet such requirements either, and these actually perform worse than the implementation analyzed in this study. This is actually the case for the proposals that transmit the first copy of a packet in dedicated resources for the UEs. These resources can be reserved using grant-based scheduling (such as in [14]) or semi-persistent scheduling (such as in [13]). Grant-based scheduling requires the UE to send an SR to the BS, and wait for the BS to reply with a grant message. The exchange of these messages between the UE and the BS is illustrated in Figure 8 This handshaking generates a non-negligible *T_total_* latency that is equal to:

*T_total_* = 2 *T*_*L*1/*L*2_ + *T_align_* + 2 *T_proc_* + 3 *T_tx_* = 2.3 ms (27)

where *T_L_*_1*/L*2_ is the *L*1/*L*2 processing latency at the BS and the UE, *T_align_* is the alignment latency (the alignment latency is the time elapsed from the moment the UE is ready to transmit to the actual time the transmission starts), *T_proc_* is the processing latency (this latency represents the latency between the reception of the SR and the transmission of the grant message), and *T_tx_* is the time required to transmit the SR and grant messages. Following [24], we consider *T_L_*_1*/L*2_ = *T_align_* = *T_tx_* = 1 TTI, and *T_proc_* = 2.33 TTI. These values are a best-case scenario, since they represent reduced processing times that can be achieved with 3GPP Release 15 compared to Release 14. Equation (27) shows that the total latency (2.3 ms) introduced by the grant-based scheduling process to assign dedicated resources to UEs is higher than the latency achieved with the 5G NR grant-free scheduling implementation analyzed in this study. For example, Figure 7 shows that this implementation can guarantee latency levels below 1 ms (this latency is guaranteed with a reliability up to 1–10^−5^ when *K* = 4, *λ* = 0.1 packets, *U* = 6, and ∆*t* = ∆*t_avg_*).

The alternative to grant-based scheduling is Semi-Persistent Scheduling (SPS). In this case, UEs are assigned dedicated resources for a period of time. During this period, UEs can utilize the resources without requesting permission from the BS. This avoids the latency introduced by grant-based scheduling. However, semi-persistent scheduling inefficiently utilizes the radio resources when the traffic is aperiodic. This is the case, because it is not possible to predict when UEs will need resources. To illustrate this effect, let us consider a scenario with *N* = 300 users that generate aperiodic traffic (*λ* = 0.1 packets). We shall then suppose that users request a maximum latency of 1 ms and a reliability level equal to 1–10^−5^. Satisfying this demand requires reserving 300 RBs (one per UE) in a 1 ms time windowA lower number of resources would be necessary if traffic was periodic and we could estimate when each UE would require resources for their transmission. In this case, several UEs could share the same RB if they generate their packets at different time instants. This would reduce the total number of RBs necessary to serve all users. This is not possible in the case of aperiodic traffic, since we cannot predict when a UE would need radio resources. Figure 7 shows that our implementation of 5G NR grant-free scheduling with 4-repetitions and shared resources can support 300 UEs (with their latency and reliability demands) with only 48 RBs in a time window of 1 ms. This is 84% less radio resources than if we reserve dedicated resources per UE (with aperiodic traffic) for their first transmission using semi-persistent scheduling. These results clearly show that the implemented 5G NR grant-free scheduling with shared resources can better support URLLC applications with aperiodic traffic and stringent communication requirements than other existing proposals. However, the conducted analysis (e.g., Figure 7) has also shown that new solutions will be needed to guarantee very demanding reliability and latency levels such as those foreseen for some URLLC services in 3GPP Release 16.

## 6. Conclusions

This paper has analyzed the capacity of 5G NR grant-free scheduling to support URLLC services with strict reliability and latency levels such as those demanded by Industry 4.0. The study has focused on aperiodic or sporadic traffic and an implementation of 5G NR grant-free scheduling with *K*-repetitions and shared radio resources. This implementation has been chosen, since sharing radio resources is an attractive option for aperiodic traffic. In addition, the *K*-repetitions scheme can combat possible packet collisions between UEs that share radio resources. This study has analyzed the reliability and latency levels that can be achieved with existing 5G NR grant-free scheduling with shared radio resources as a function of the number of UEs, the number of reserved radio resources, and the number of replicas *K*. To this aim, this study has derived analytical expressions that quantify the exact probability of collision with packets transmitted by other UEs, and the impact of self-collisions. It is important to emphasize that this study is the first one that has evaluated the impact of self-collisions. Packet collisions and self-collisions have then been taken into account to derive analytically the reliability that can be achieved by existing 5G NR grant-free scheduling with shared resources. The derived analytical expressions have been validated against simulations. These expressions are a valuable contribution to the community, since they can be easily utilized to evaluate 5G NR grant-free scheduling.

This study has demonstrated that current 5G NR grant-free scheduling solutions cannot guarantee high reliability levels with strong latency requirements. This is partly due to the fact that strong latency requirements limit the number of replicas *K* that can be transmitted. In addition, self-collisions have a non-negligible impact that even limits the reliability that can be achieved when *K* increases. The impact of self-collisions also increases with the latency requirements. The obtained results demonstrate that new solutions are necessary for 5G NR grant-free scheduling to be able to support applications with stringent URLLC latency and reliability requirements under the presence of aperiodic traffic. In particular, the transmission of *K* replicas per packet might be inadequate to support aperiodic traffic with very low latency levels due to the impact of self-collisions. Consequently, other approaches should be designed to minimize collisions between UEs sharing radio resources. This study has shown that these new solutions cannot be based either on grant-based or semi-persistent scheduling. Grant-based scheduling introduces additional latency due to the exchange of messages between the UEs and the BS for assigning the radio resources. Semi-persistent scheduling with dedicated resources inefficiently utilizes the available resources when considering dedicated resources and aperiodic traffic. Innovative grant-free scheduling solutions are hence necessary to meet the URLLC requirements identified for 3GPP Release 16 and beyond. This could include, for example, the use of sensing mechanisms or full duplex techniques that can reduce packet collisions.

## Figures and Tables

**Table 1:** *P_sc_* for *L* = 1 ms.

*K*	*Λ* = 0.1 Packets		*Λ* = 1 Packet	
	∆*t* = ∆*t*_1_	∆*t* = ∆*t*_2_	∆*t* = ∆*t*_1_	∆*t* = ∆*t*_2_
4	0	1.25 × 10^−5^	0	1.25 × 10^−4^
6	5.00 × 10^−5^	6.25 × 10^−5^	5.00 × 10^−4^	6.25 × 10^−4^
8	9.99 × 10^−5^	1.13 × 10^−4^	9.99 × 10^−4^	1.13 × 10^−3^

## References

[1] 3GPP (2018). Technical Specification Group Services and System Aspects.

[2] 3GPP (2018). Technical Specification Group Radio Access Network.

[3] Klessig H., Ashraf S.A., Almeroth B., Riedel I., Puschmann A., Elste T., Simsek M., Schulz P., Matthe M., Fettweis G. (2017). Latency Critical IoT Applications in 5G: Perspective on the Design of Radio Interface and Network Architecture. IEEE Commun. Mag..

[4] 5G Alliance for Connected Industries and Automation (5G-ACIA) (2019). 5G for Connected Industries and Automation.

[5] Popovski P., Nielsen J.J., Stefanovic C., De Carvalho E., Ström E., Trillingsgaard K.F., Bana A.-S., Kim D.M., Kotaba R., Park J. (2018). Wireless Access for Ultra-Reliable Low-Latency Communication: Principles and Building Blocks. IEEE Netw..

[6] 3GPP (2016). Technical Specification Group Radio Access Network.

[7] 3GPP (2018). Technical Specification Group Radio Access Network.

[8] Berardinelli G., Mahmood N.H., Abreu R., Jacobsen T., Pedersen K., Kovacs I.Z., Mogensen P. (2018). Reliability Analysis of Uplink Grant-Free Transmission Over Shared Resources. IEEE Access.

[9] 3GPP (2018). Discussion on Configured Grant for NR-U.

[10] Li Z., Uusitalo M.A., Shariatmadari H., Singh B. 5G URLLC: Design Challenges and System Concepts. Proceedings of the 2018 15th International Symposium on Wireless Communication Systems (ISWCS).

[11] 3GPP (2018). Technical Specification Group Radio Access Network.

[12] Wu Y., Zhang L., Wang C., Chen Y. Performance Evaluation of Grant-Free Transmission for Uplink URLLC Services. Proceedings of the 2017 IEEE 85th Vehicular Technology Conference (VTC Spring).

[13] Kotaba R., Manchon C.N., Balercia T., Popovski P. (2018). Uplink Transmissions in URLLC Systems with Shared Diversity Resources. IEEE Wirel. Commun. Lett..

[14] Abreu R., Mogensen P., Pedersen K.I. Pre-Scheduled Resources for Retransmissions in Ultra-Reliable and Low Latency Communications. Proceedings of the 2017 IEEE Wireless Communications and Networking Conference (WCNC).

[15] Singh B., Tirkkonen O., Li Z., Uusitalo M.A. (2018). Contention-Based Access for Ultra-Reliable Low Latency Uplink Transmissions. IEEE Wirel. Commun. Lett..

[16] Jacobsen T., Abreu R., Berardinelli G., Pedersen K., Mogensen P., Kovacs I.Z., Madsen T.K. System Level Analysis of Uplink Grant-Free Transmission for URLLC. Proceedings of the 2017 IEEE Globecom Workshops (GC Wkshps).

[17] 3GPP (2017). Grant-Free Transmission for UL URLLC.

[18] 3GPP (2018). Enhancement of Uplink Grant-free transmission for NR URLLC.

[19] 3GPP (2018). Enhancement of Configured Grant for NR URLLC.

[20] 3GPP (2018). Technical Specification Group Radio Access Network.

[21] 3GPP (2018). Technical Specification Group Radio Access Network.

[22] 3GPP (2018). Technical Specification Group Radio Access Network.

[23] Bennis M., Debbah M., Poor H.V. (2018). Ultrareliable and Low-Latency Wireless Communication: Tail, Risk, and Scale. Proc. IEEE.

[24] 3GPP (2017). Evaluation of Latency in LTE.

